# Establishment of Patient-Derived Organoids for Pediatric Cancer Research

**DOI:** 10.3390/cancers18091465

**Published:** 2026-05-02

**Authors:** Muhammad Younis, Tarlan Arjmandi, Mohammad Haque, Katherine McClain, Thussenthan Walter-Angelo, Franklin Back, Divya Gandra, Abigail Moore, Chandrika Behura, Vladimir S. Spiegelman, Hong-Gang Wang, Sinisa Dovat, Jeremy Hengst, Giselle Saulnier Sholler

**Affiliations:** 1 Department of Pediatrics, Hematology/Oncology, College of Medicine, Pennsylvania State University, Hershey, PA 17033, USA; myounis@pennstatehealth.psu.edu (M.Y.); tarjmandi@pennstatehealth.psu.edu (T.A.); mhaque@pennstatehealth.psu.edu (M.H.); kmcclain@pennstatehealth.psu.edu (K.M.); twalterangelo@pennstatehealth.psu.edu (T.W.-A.); fback@pennstatehealth.psu.edu (F.B.); dgandra@pennstatehealth.psu.edu (D.G.); amoore13@pennstatehealth.psu.edu (A.M.); cgowda2@pennstatehealth.psu.edu (C.B.); vspiegelman@pennstatehealth.psu.edu (V.S.S.); hwang3@pennstatehealth.psu.edu (H.-G.W.); sdovat@pennstatehealth.psu.edu (S.D.); 2 Pediatric Hematology and Oncology Division, Pennsylvania State University, Golisano Children’s Hospital, Hershey, PA 17033, USA

**Keywords:** pediatric cancer, patient derived organoids, preclinical model

## Abstract

**Background/Objectives**: Pediatric cancers are disorders of dysregulated development driven largely by genomic and epigenetic alterations. Precisely modeling these developmental differences is essential for understanding the unique biology of childhood cancers. Patient-derived organoids (PDOs) offer a powerful in vitro platform that recapitulates tumor heterogeneity, plasticity, microenvironment (including immune cells) and disease-relevant features. **Methods**: Here, we describe a step-by-step protocol for the establishment of PDOs from cells derived from pediatric brain tumors and extracranial solid tumor biopsies and bone marrow aspirates, including tumor processing, organoid culture/subculture, and cryopreservation. **Results**: Furthermore, we present the use of PDOs for further experimental analysis such as fluorescence imaging, Western blotting, flow cytometry, and immunohistochemistry (IHC) to investigate the underlying pathophysiology of tumorigenesis. **Conclusions**: Expanding the application of organoids to childhood malignancies holds exceptional promise for elucidating pediatric tumor biology and advancing therapeutic strategies, representing the long-overdue convergence of technology and clinical need.

## Simple Summary

Childhood cancer arises from disrupted developmental processes driven by genetic and epigenetic changes, making them fundamentally different from adult malignancies. Understanding this unique biology requires models that faithfully capture the complexity of pediatric cancer. Patient-derived organoids (PDOs) have emerged as a transformative tool in this space, preserving the heterogeneity, plasticity and microenvironmental features of the original tumor in the laboratory setting. This protocol provides research through the practical steps of building PDOs from pediatric tumors, solid tumor biopsies and bone marrow aspirates. These living models are then applied to a range of analytical techniques, including florescence imaging, Western blot analysis, flow cytometry and IHC, to unravel how these tumors develop and behave. Bringing organoid technology into pediatric oncology represents a meaningful step towards accelerating both our biological understanding and the development of better treatments for children with cancer.

## 1. Introduction

Three-dimensional (3D) culture systems are now important tools for studying human tissues and diseases in the lab. Organoids are a special type of 3D structure that can form on their own from primary tissue or stem cells and retain many key features of the original tissue, such as its structure, cell types, and functions [1,2,3]. Unlike traditional two-dimensional (2D) cell cultures, organoids keep a natural 3D layout, allow cells to communicate with each other, and include different cell lineages, which makes them very useful for studying complex processes like development, tissue balance, immune cell interactions, vascularization (angiogenesis, hypoxia) [4,5] and how diseases develop over time. In cancer research, organoids make it possible to study how tumors grow in conditions that are much closer to what happens inside the body [6,7].

Conventional 2D cancer cell lines have been very useful for learning basic molecular mechanisms, but they lack many key features of real tumors. Cells grown in 2D do not have a realistic tissue structure, receive the same amount of nutrients and oxygen, and often lose their specialized characteristics over time. Because of this, 2D cultures do a poor job of reflecting the true diversity of tumor cells, their organization, and their interactions with the surrounding environment. These problems are especially important when studying how tumors grow, how cell lineages change, and how cancers respond to treatments, since all these processes depend heavily on 3D structure and cell–cell communication. Three-dimensional models, such as organoids, help solve these issues by allowing complex multicellular organization and giving a more accurate picture of how tissues behave [8,9,10].

The need for better modeling systems is especially important for studying pediatric cancer [11]. Unlike most adult cancers, which usually develop after many genetic mutations build up over time, childhood tumors often start when normal developmental programs are disturbed [12]. Pediatric cancers typically have fewer genetic mutations overall and are more strongly driven by changes in epigenetic regulation, developmental signaling pathways, and lineage-specific cancer-causing events [13]. These tumors form in tissues that are still developing and often show strong cellular plasticity and clear hierarchical organization. As such, pediatric tumors are biologically different from adult cancers and cannot be accurately studied using models that were designed for adult epithelial tumors [14].

The pediatric tumors examined in this study include neuroblastoma (NB), Ewing sarcoma (EWS), diffuse intrinsic pontine glioma (DIPG), osteosarcoma (OS), medulloblastoma (MBL), rhabdoid tumors such as atypical teratoid/rhabdoid tumors (ATRT), Rhabdomyosarcoma (RB) and Hepatoblastoma (HP), which are all aggressive childhood cancers with strong links to development. Neuroblastoma comes from neural crest-derived sympathetic precursor cells and shows high cellular diversity and flexible differentiation [15,16]. Ewing sarcoma is caused by cancer-promoting fusion proteins that change gene expression and epigenetic patterns in mesenchymal progenitor cells [17]. DIPG is a highly aggressive brain tumor that forms in the developing brainstem and is marked by major epigenetic disruption [18]. Osteosarcoma develops from osteoblast lineage cells during periods of rapid bone growth, while medulloblastoma originates from cerebellar progenitor cells and includes several molecular subgroups tied to developmental signaling pathways. Rhabdoid tumors, including ATRTs, result from the loss of chromatin remodeling components and show very undifferentiated cell states. Although these cancers come from different tissues, they share key challenges, such as having few adequate lab models and difficulty in preserving their diverse cell populations in standard culture systems [13].

Even with progress in cancer models, most systems have been designed mainly for adult cancer. Organoid technology is widely used to study adult solid tumors, where it can replicate important features like tumor structure, genetic changes, and both between- and within-tumor diversity. In comparison, organoid models for pediatric solid tumors are much less developed. This is partly because of technical difficulties, limited access to patient samples, and the wide biological diversity of childhood cancers, many of which do not come from epithelial tissues. Due to these issues, there is still a strong need for realistic, scalable lab models that truly reflect the developmental background and cellular complexity of pediatric tumors.

Establishing patient-derived organoids (PDOs) from pediatric tumors offers a promising strategy to address this gap. By maintaining a three-dimensional structure and enabling the coexistence of multiple tumor cell populations, PDOs provide a platform to study tumor growth, differentiation states, cellular heterogeneity, and immune invasion in a controlled environment. Importantly, PDOs allow direct investigation of tumor-intrinsic properties without the need for long-term adaptation or immortalization, which can alter tumor biology [19]. When combined with downstream analytical approaches, PDOs can be used to characterize tumor architecture, identify distinct cellular populations, and assess expression of lineage- and disease-specific markers [20].

**Figure 1:**
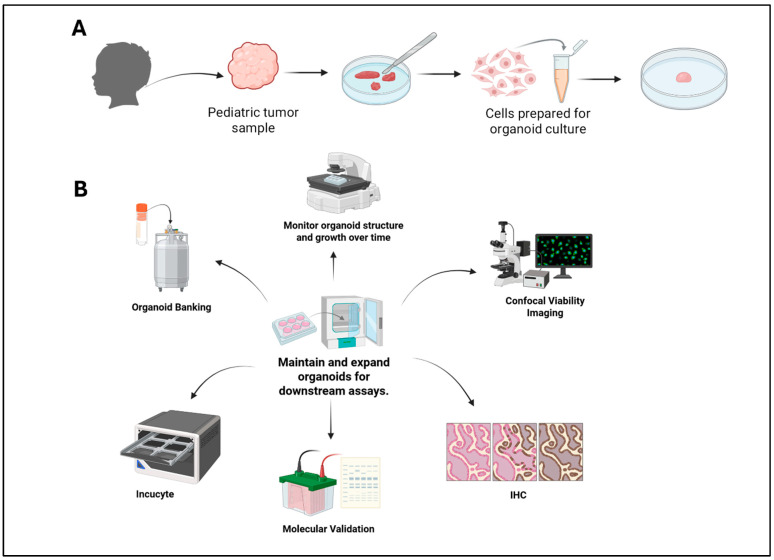
Development of patient organoids. Patient samples (tumor biopsies, bone marrow aspirates) were processed and cultured into organoid models (**A**). PDOs could be used for further analysis such as drug screening, fluorescence imaging, IHC, immunoblotting, live cell imaging, and long-term storage (**B**).

Previously, our lab reported the successful establishment and use of cell lines from various types of pediatric cancer research such as neuroblastoma [21,22], Ewing sarcoma [17], medulloblastoma [23], and alveolar rhabdomyosarcoma [24]. In this study, we generated patient-derived organoids from pediatric solid tumor biopsies from primary tumor sites or from metastatic disease in bone marrow aspirates, including NB, EWS, DIPG, OS, ATRT, MBL, RB, and HP, that reflect the specific environment from which tumor cells were derived, contain biological diversity, and ensure that our models more accurately represent the patients’ disease state. Tumor samples were obtained and processed to establish PDO cultures, which were subsequently expanded under defined conditions. Organoid growth and morphology were assessed using live imaging approaches, while tissue organization and marker expression were evaluated using hematoxylin and eosin staining and immunohistochemistry. In parallel, flow cytometry was performed to quantify distinct cellular populations and examine differences in marker-defined cell states within the organoids. Western blot analysis was additionally used to assess the expression of key tumor- and lineage-associated proteins across individual PDO models. Together, these complementary approaches enabled a systematic characterization of pediatric tumor PDOs, providing a foundation for studying growth behavior, cellular composition, and tumor heterogeneity in a physiologically relevant 3D system (Figure 1).

## 2. Materials and Methods

### 2.1. Patient Samples and Cell Lines

Subjects were enrolled onto Beat Childhood Cancer Research Consortium clinical trials (NCT04715178, NCT03581240). PDOs were established from different pediatric cancer patient cell lines including neuroblastoma (SL00712-106128), Ewing sarcoma (SL02370-199764), osteosarcoma (SL02398-201129), rhabdomyosarcoma (SL03361-198551), diffuse intrinsic pontine glioma (SL01469-142071), medulloblastoma (SL02229-186886), hepatoblastoma (SL02433-201221), embryonal tumor with multilayered rosettes (SL00895-200834), NB bone marrow aspirate (SL02651-203684), EWS bone mane marrow aspirate (SL02164-203473) and mouse neuroblastoma (9464D-M2) provided by Dr. Vladimir Spiegelman’s lab. Models generated from this research can be made available to researchers with the establishment of a material transfer agreement (MTA) within the Beat Childhood Cancer Research Consortium network.

### 2.2. Reagents

DMEM-F12 (Gibco; Waltham, MA, USA; Cat # 12587010), Glutamax (Gibco Cat # 12587010), Anti:Anti (100X) (Gibco Cat # 265249), TrypLe Express (Gibco Cat # 2-604-039), Matrigel (Corning; Corning, NY, USA; Cat # 354234), Basic fibroblast growth factor (bFGF) (PeproTech; Cranbury, NJ, USA; Cat # 10018B250UG), B27 (Without Vitamin A) (Gibco Cat # 12587010), Epidermal growth factor (EGF) (Gibco Cat # PHG0311L), N-2 (Gibco Cat # 17502048), BIT 9500 Serum Substitute (STEMCELL; Cambridge, MA, USA; Cat # 9500), MEM non-essential amino acids (Gibco Cat # 12587010), β-mercaptoethanol (Gibco Cat # 12587010), leukemia inhibitory factor (LIF) (Millipore, Billerica, MA, USA; Cat # 12587010), ROCK (Rho kinase) inhibitor (Millipore Cat # S1049), Primocin (Invivogen; San Diego, CA, USA; Cat # ant-pm-05), Dispase II (STEMCELL Catalog # 07913), Collagenase type IV (Gibco Cat # 17104-019), Cyto3D^®^ Live-Dead Assay Kit (Biowell; Westland, MI, USA; Cat No.: BM01), blocking reagent (BioLegend; San Diego, CA, USA; #422302) human CD45 (Invitrogen; Carlsbad, CA, USA; Cat # 58-0459-42), CD3 (Invitrogen Cat # 16-0037-81), B220 (BioLegend Cat# 103206) and CD11b (BioLegend Cat # 101241), FACS stain buffer (BD Pharmingen; San Diego, CA, USA; Cat # 554657).

### 2.3. Tissue Collection and Handling

#### 2.3.1. Tissue/Bone Marrow Aspirate Preparation

Primary tumor specimens were collected in cold Macs^®^ TSS and cultured in RPMI 1640 or Advanced DMEM/F12 basic medium. Using a 5 mL sterile pipette, carefully remove the tumor tissue from the original non-vented cryovial and place it on the 100 mm Petri dish. The tumor or bone core were minced (without pulling the tissue) into ~0.5–1 mm^3^ fragments using sterile scalpels or fine scissors. In a culture dish, cells were allowed to sit in an incubator in the appropriate culture media for 20–25 min and then immediately transferred to a T25 flask. When tissue quantity permitted, material was allocated for primary culture, organoid generation, animal implantation, and molecular analyses; excess tissue was snap-frozen in liquid nitrogen. For bone marrow aspirate samples, all reagents, materials, and tools (including forceps) were autoclaved and disinfected with 70% ethanol prior to use. Two tubes were labeled as BMA and Flow Through (FT). Using sterile forceps, a filter was placed into the BMA tube, and right (R) and left (L) samples were passed through the filter without direct contact. After filtration, the filter was transferred to the FT tube using sterile forceps. The BMA tube was diluted 1:1 with cold sterile 1× PBS. The filter in the FT tube was washed with 15 mL of cold 1× PBS to remove red blood cells. The filter was transferred to a 10 cm culture dish containing appropriate media (K-DISH). Cells were gently washed off the filter into the dish, and the dish was placed in the incubator. The FT tube volume was adjusted to 50 mL by adding 35 mL ammonium chloride-based RBC lysis solution and incubated at 4 °C for at least 30 min. A SepMate conical tube was prepared with 15 mL Ficoll added through the bottom port. The diluted BMA sample was carefully layered onto the Ficoll to maintain gradient separation. Samples were centrifuged for 20 min at room temperature at 1200× *g* with the brake on. Following centrifugation, the mononuclear cell layer was identified and transferred to a new 50 mL conical tube labeled MNC Tube, then brought to 50 mL with 1× PBS. The MNC and FT tubes were centrifuged for 5 min at room temperature, followed by aspiration of supernatants and washing with 50 mL 1× PBS. MNCs were resuspended in 10 mL cold 1× PBS, counted according to SOP guidelines, and then brought to final volume with additional PBS. The FT pellet was resuspended in media from the K-DISH and returned to the dish. MNCs were resuspended in 800 μL freezing media (FBS + 10% DMSO), transferred to cryovials, placed in a controlled-rate freezing container (Mr. Frosty), and stored at −80 °C. KD/FT cultures were maintained and used for organoid development.

#### 2.3.2. Enzymatic Dissociation

Minced tissue was transferred to conical tubes containing collagenase IV and digested at 37 °C with intermittent mechanical trituration until fragments were dispersed. Digested material was gently washed to remove adipose tissue and blood components, followed by centrifugation (300–500× *g*). Residual connective tissue was further dissociated using TrypLE Express when required. Red blood cells were removed using ACK lysis buffer when visible.

#### 2.3.3. Cell Recovery

Cells and small tissue fragments were washed, pelleted, and resuspended in a basic medium (RPMI/DMEM). The final pellet was used immediately for downstream culture.

#### 2.3.4. Primary Cell Culture Establishment

Cells were incubated at 37 °C for 25 min to allow preferential fibroblast attachment. The supernatant containing non-adherent tumor cells was transferred to fresh vented T25 flasks. Cultures were maintained at 37 °C in a humidified incubator with 5% CO_2_ and monitored routinely for growth and morphology.

#### 2.3.5. Organoid Initiation

A subset of freshly isolated cells was immediately used to establish three-dimensional organoid cultures as described separately.

### 2.4. Organoid Culture

#### 2.4.1. Initiation of Organoids

Patient-derived organoids were seeded in 24-well plates and put at 37C before use for 15–20 min. For surgical specimens, a fraction of digested cells was plated in 60 mm dishes containing high-glucose DMEM supplemented with 20% FBS and 1% anti-anti to establish parallel 2D cultures. Matrigel matrix (Corning) was thawed overnight on ice at 4 °C and always kept on ice to prevent premature polymerization. Cell pellets were resuspended in ice-cold Matrigel at a density of ~30–50 µL (contains 1 × 10^4^) per well, mixed gently, and plated slowly into the center of each well. Plates were incubated at room temperature for 5 min and then transferred to a 37 °C incubator for 10 min to allow matrix solidification. Solidified droplets were overlaid with 500 µL of organoid culture media supplemented with 1 Gultamax, 1% penicillin/streptomycin, 40ng/mL basic fibroblast growth factor (bFGF), 20 ng/mL epidermal growth factor (EGF), 1% n-2, 10% BIT, 1% MEM non-essential amino acids, 50 μM β-mercaptoethanol and 500 μ/mL LIF per well and incubated for 1–3 weeks, depending on tumor type, cellular proliferation rates, and sample quality. Medium was added or partially replaced every 4 days. Organoid formation was typically observed within 1–3 weeks. Once robust organoid growth was established, one vial per case was cryopreserved immediately when sufficient cell numbers were available. Cultures were then expanded to generate material for long-term storage (≥5 vials). Tumor-derived organoids generally exhibited slower growth kinetics and displayed multilayered or solid morphologies.

#### 2.4.2. Maintenance and Passaging of Organoids

Organoid cultures were fed or medium was partially replaced every 2–3 days. Cultures were monitored closely to avoid overgrowth, indicated by yellowing of the medium. Organoids were passaged every 6–7 days at a split ratio of 1:4 to 1:6.

#### 2.4.3. Subculture Procedure

Approximately half of the culture medium was carefully aspirated. Organoids from 3 to 4 wells were collected into a microcentrifuge tube and mechanically disrupted by pipetting 10 times using a 1 mL pipette tip. Samples were centrifuged at 600–700× *g* for 3 min, and supernatant was removed without disturbing the organoid–matrix pellet. Dispase II (5 U/mL) was added to digest the Matrigel matrix, followed by incubation at 37 °C for 12–15 min with intermittent trituration. After centrifugation (600–700× *g*, 3 min), the supernatant was removed and pellets were resuspended in 500 µL TrypLE Express. Samples were incubated for 5 min at 37 °C, pipetted repeatedly to fragment organoids, and neutralized with cold basic medium. Cells were washed by centrifugation, and all residual medium was removed using a fine pipette tip. Pellets were resuspended in ice-cold Matrigel matrix and replated at 30–50 µL per well in low-attachment 24-well plates. Plates were left at room temperature for 5 min, transferred to 37 °C for 10 min, and overlaid with 500 µL expansion medium. Medium was replaced every 2–3 days, and cultures were passaged every 6–7 days. All plates were UV-sterilized and preheated at 37C prior to use.

#### 2.4.4. Cryopreservation of Organoids

Freezing vials were prepared with 1.3 mL freezing medium and kept on ice. Organoids were processed through subculture up to the final wash step and resuspended in freezing medium (200 µL per well of confluent organoids). Cell suspensions were transferred to freezing vials, gently mixed, and stored temporarily at −80 °C and then transferred to liquid nitrogen. Freezing was performed at a ratio of one well of confluent organoids per vial.

#### 2.4.5. Thawing of Frozen Organoids

Frozen organoids were thawed using standard cell line procedures and plated at a ratio of one vial per 2–3 wells. Cultures were maintained for 6 days in organoid media. After 6 days, early-passage organoids (≤P3) readily attached and proliferated when transferred to 2D culture, whereas late-passage organoids (>P3) exhibited minimal attachment.

#### 2.4.6. Western Blot Analysis

PDOs were collected and dissociated to single cells by using Dispase II, followed by TrypLE Express, and resuspended in RIPA buffer supplemented with Halt™ Protease Inhibitor Cocktail (Cat# 78425). Immunoblotting was performed according to the previously reported method [25,26]. In short, protein concentrations were determined and equal volumes were separated on 10–12% SDS-PAGE and transferred to the PVDF membrane. Subsequently, membranes were incubated with primary antibody N-myc (Cell Signaling Technologies (CST; Danvers, MA, USA); Cat # 84406S), PCNA (CST; Cat # 13110S), vimentin (CST; Cat # 63269S), SOX2 (CST; Cat # 3579S), Nanog (CST; Cat # 4903S) and GAPDH (CST; Cat # 5174S) overnight at °C. The membrane was rinsed with TBST before being treated with secondary antibody. The membranes were then washed three times with TBST, and an enhanced chemiluminescence (ECL) kit was used to detect proteins.

### 2.5. Flow Cytometry

Flow cytometric analysis was performed to investigate the presence of immune cells to elucidate the heterogenous nature of the organoid that mimics the tumor in humans. PDOs were dissociated to obtain single cells as described above. After centrifugation, cells were washed with phosphate-buffered saline (PBS) containing 2% fetal bovine serum (FBS). Then, 1 × 10^6^ cells were incubated with Fc receptor blocking reagent (BioLegend Cat # 422302) for 10 min at 4 °C, followed by staining with different fluorochrome-conjugated monoclonal antibodies against human CD45 (Invitrogen Cat # 58-0459-42), CD3 (Invitrogen Cat # 16-0037-81), B220 (BioLegend Cat # 103206) and CD11b (BioLegend Cat # 101241), for 30 min at 4 °C in the dark. After staining, cells were washed twice with PBS/2% FBS and resuspended in FACS stain buffer (BD Pharmingen Cat # 554657). After staining, cells were washed twice with PBS/2% FBS and resuspended in FACS stain buffer. Data were acquired on a BD LSR Fortessa flow cytometer and analyzed using FlowJo v10. For gating strategy, human CD45 was gated first and then T cells, B cells and macrophage were gated from CD45. For intracellular staining, cells were fixed and permeabilized using the intracellular fixation and permeabilization Buffer Set (eBioscience; San Diego, CA, USA; Cat # 88-8824-00) according to the manufacturer’s instructions. Cells were then stained with antibodies against PhoX2B (vendor and catalog number) for 30 min at 4 °C, washed, and analyzed by flow cytometry.

### 2.6. HE and IHC Staining

PDOs were fixed in 10% formalin for 24 h and embedded in paraffin. The paraffin block was serially sectioned at a thickness of 5 um and used for H&E and IHC staining of ki-67 was performed for NB and MBL organoids. Formalin-fixed paraffin-embedded organoid sections were processed on a Leica BOND system. Slides were dewaxed using BOND Dewax Solution followed by 100% alcohol and BOND Wash Solution under a pre-programmed protocol. Antigen retrieval was performed using BOND Epitope Retrieval ER2 solution for 20 min. Endogenous peroxidase activity was blocked for 5 min using the Refine Detection Kit Peroxide Block, followed by washing with BOND Wash Solution. Sections were blocked with normal goat serum or animal-free blocking solution for 20 min, then incubated with the Ki-67 primary antibody diluted in SignalStain^®^ Antibody Diluent for 30 min. After washing, rabbit antibodies were incubated with a post-primary rabbit linker for 10 min, followed by secondary detection using the Refine Detection Kit Polymer for 10 min. Signal visualization was achieved using Mixed DAB Refine solution, followed by hematoxylin counterstaining for 5 min. Slides were washed, dehydrated offline through graded ethanol, and mounted with coverslips using SignalStain^®^ Mounting Medium.

### 2.7. Cell Viability Analysis

After growing the NB (9464D-M2) organoids for 7–10 days, we performed a cell viability assay by using Cyto3D as per the manufacturer’s instructions as below. Bring the Cyto3D^®^ Live-Dead Assay Kit to room temperature. Add 2 µL of Cyto3D^®^ reagent to every 100 µL total volume in a well. (Note: Adjust the volume of Cyto3D^®^ reagent according to the total Matrigel volume and medium ratio 1:1. For example, for 3D cell culture, 50 µL Matrigel + 50 µL cover medium = total volume of 100 µL.) Incubate the cells at 37 °C for 10–20 min. Then, we imaged live and dead cells in organoids by using the Sartorius Incucyte system.

### 2.8. Measurement of Organoid Size

Organoids derived from bone marrow aspirates of neuroblastoma (NB) and Ewing sarcoma (EWS) were monitored for growth over time. Brightfield images were captured on day 1, day 5, and day 10, and organoid diameters were measured using ImageJ software (National Institutes of Health, Bethesda, MD, USA). For each condition, multiple organoids were analyzed, and the average size was calculated. Statistical analysis was performed to compare organoid sizes across time points, with day 1 used as the reference group. Data are presented as mean ± standard deviation (SD), and statistical significance was determined using appropriate tests as indicated in the figure legends.

### 2.9. Statistical Analysis

Quantitative data are presented as mean ± standard error of the mean (SEM). Statistical analyses were conducted using GraphPad Prism software (Version 11.0.0). Comparisons between two groups were performed using an unpaired two-tailed Student’s *t*-test, while multiple group comparisons were analyzed using one-way analysis of variance (ANOVA) followed by appropriate post hoc tests. Densitometric analysis of Western blot bands and organoid size measurements was included in the statistical evaluation. A *p*-value of less than 0.05 was considered statistically significant.

## 3. Results

**Figure 2:**
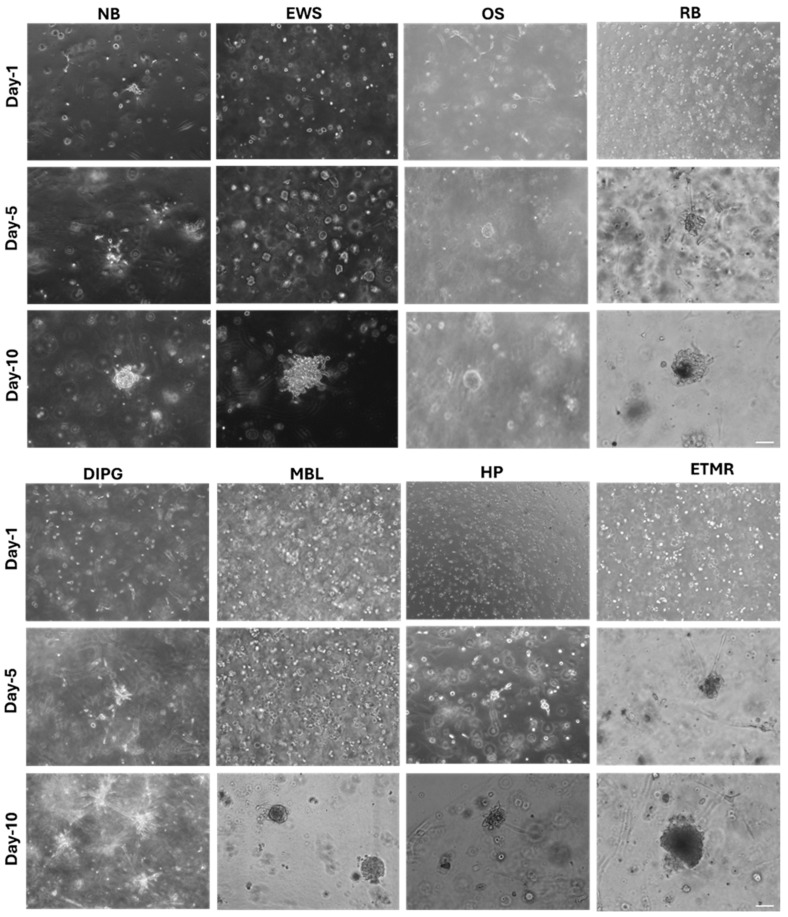
Establishment and growth dynamics of patient-derived organoid (PDO) models from pediatric tumors. Tumor tissues were processed using standard dissociation protocols, and derived cells were cultured to establish PDO models across multiple pediatric malignancies, including neuroblastoma (NB), Ewing sarcoma (EWS), osteosarcoma (OS), rhabdomyosarcoma (RB), diffuse intrinsic pontine glioma (DIPG), medulloblastoma (MBL), hepatoblastoma (HP), and embryonal tumor with multilayered rosettes (ETMR). Organoid formation and morphological development were monitored over time and imaged on day 1, day 5, and day 10 using an EVOS inverted microscope at 10× magnification. Representative images illustrate progressive organoid growth and structural organization across time points. Scale bar: 100 µm.

We generated eight different kinds of PDOs corresponding to eight different patients including neuroblastoma (NB), Ewing sarcoma (EWS), osteosarcoma (OS), rhabdomyosarcoma (RB), DIPG, medulloblastoma (MBL), hepatoblastoma (HP), and ETMR (Figure 2). PDOs were generated from 5 × 10^3^, 1 × 10^4^ and 5 × 10^4^ cells embedded in 50 μL of Matrigel and cultured for a maximum of 2 weeks without passaging. We observed efficient cell growth at all cellular concentrations tested, and we chose 1 × 10^4^ cells/droplet of Matrigel for the following experiments of the study. The formation and growth of the PDOs were monitored for two weeks and three-dimensional morphology was imaged at days 1, 5 and 10 (Figure 2). The sizes of patient-derived organoids (PDOs) were quantified using ImageJ software (Version 1.54S), demonstrating measurable growth characteristics across samples (Figure S1). In order to investigate the possibility of expanding and maintaining the organoids as reservoirs of patient-derived material, we performed tests to evaluate the ability of PDOs to be cryopreserved and expanded; PDOs were dissociated to single-cell suspension and cryopreserved and thawed after 2 weeks. The frozen–thawed PDOs were able to grow and proliferate, allowing their long-term storage and retrieval.

### 3.1. PDOs Retain Proliferation, Stemness Properties and Heterogenous Cellular Composition of Tumors

**Figure 3:**
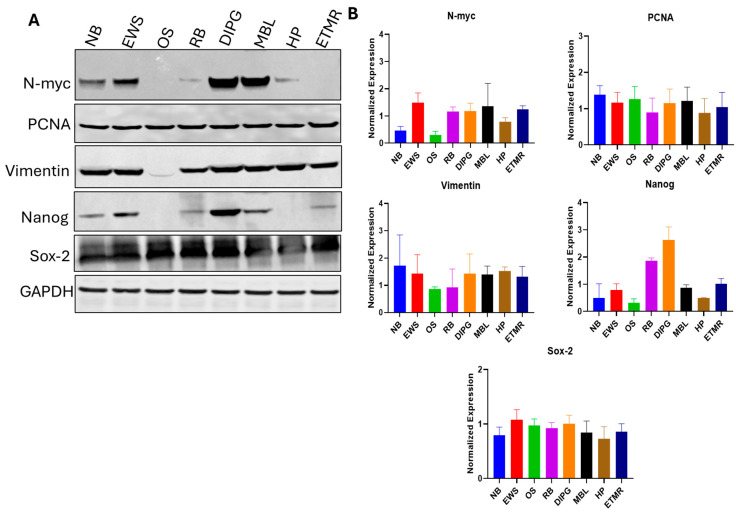
Expression of different markers in pediatric cancer. (**A**) Western blot analysis was performed to detect the expression of different proteins in organoid models. N-myc: confirms retention of tumor-specific oncogenic signaling, PCNA: assesses cell proliferation within 3D structures, Vimentin: evaluates mesenchymal features and tumor invasiveness, Nanog and Sox-2: identify cancer stem-like cell populations and assess self-renewal and tumor heterogeneity, respectively. (**B**) Band intensities were quantified using ImageJ, normalized to GAPDH, and expressed relative to control. Data represent mean ± SEM (*n* = 3).

We explored the molecular features of the PDO models, testing their functional properties by quantifying the stem cell population and tumor-initiating capability in vitro. We performed Western blot analysis of all mentioned types of pediatric cancer. First, we examined the N-myc: protein expression in all PDOs. Next, we confirmed expression of the proliferation marker PCNA in PDOs. Furthermore, we also analyzed the expression of vimentin because PDOs contain mesenchymal cells and observed that osteosarcoma PDOs express very low mesenchymal marker vimentin. Further, we examined the Nanog and sox-2 markers since they correspond to the stemness in cancer stem cells (CSCs) across various tumor types, deriving self-renewal tumorgenicity and therapy resistance. GAPDH was used as a loading control to ensure equal protein loading across samples. We also conducted densitometric analysis of protein expression levels obtained from Western blot experiments. Band intensities were quantified using ImageJ software and normalized to the loading control (Figure 3A,B).

### 3.2. Bone Marrow Aspirate-Derived Organoid

**Figure 4:**
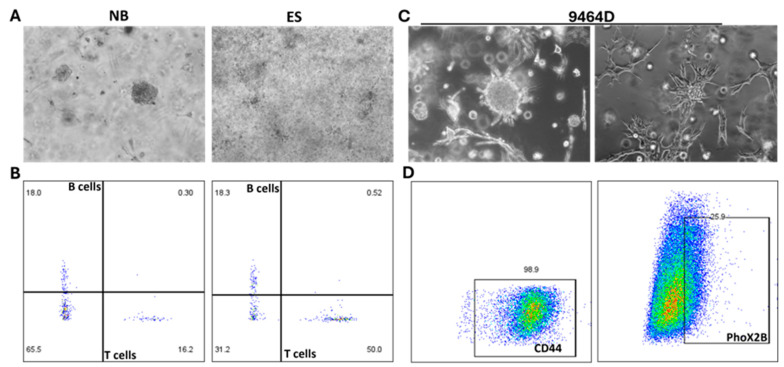
Organoid generation from bone marrow aspirate. (**A**) PDOs were established from bone marrow aspirates obtained from neuroblastoma (NB) and Ewing sarcoma (EWS) patients using standard processing and 3D culture conditions. Successful organoid formation demonstrated the feasibility of deriving tumor-representative models from bone marrow-derived material. (**B**) Immune cell populations within bone marrow-derived PDO cultures were validated by flow cytometry. Cells were analyzed for expression of human CD45 (pan-leukocyte marker), CD3 (T cells), B220 (B cells), and CD11b (myeloid cells), confirming the presence of immune subsets within the organoid microenvironment. (**C**,**D**) Further characterization was performed using the 9464D-M2 neuroblastoma model to assess tumor cell phenotype. Expression of the mesenchymal marker CD44 and the noradrenergic marker PHOX2B was analyzed, demonstrating co-existence of heterogeneous tumor cell states within the organoid system, consistent with phenotypic plasticity in neuroblastoma. Data represent mean ± SEM.

After establishing the PDOs from tumor tissues, we also generated organoids from bone marrow aspirates for NB and EWS patients (Figure 4A). The quantified PDO sizes are plotted and presented in Figure S2. After 2 weeks of growth, we performed flow cytometry analysis, investigating the presence of immune cells to elucidate the heterogenous nature of the organoid that mimics the tumor in humans. Immune cell populations were identified using human CD45 as a pan-leukocyte marker, with further characterization using CD3 (T cells), B220 (B cells), and CD11b (myeloid cells). Tumor cells were distinguished as CD45^−^ populations and further characterized using tumor-specific markers, including GD2 for neuroblastoma samples and CD99 for Ewing sarcoma samples. Isotype controls were included for all antibodies to define gating thresholds. Representative gating strategies are provided in Figure 4C,D.

### 3.3. PDOs Recapitulate Histological Tumor Characteristics

**Figure 5:**
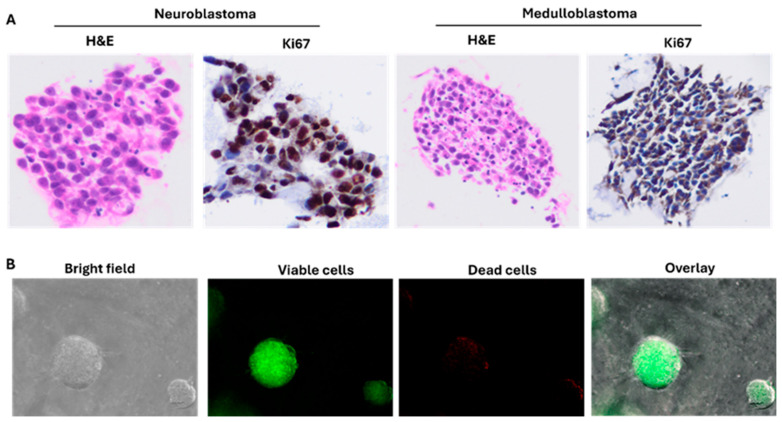
Histological and cell viability analysis of PDOs. (**A**) Histological and immunohistochemical (IHC) analyses were performed on formalin-fixed, paraffin-embedded (FFPE) PDO sections. Hematoxylin and eosin (H&E) staining was used to evaluate overall tissue architecture and organoid morphology. Immunohistochemistry was performed using Ki-67 antibody to assess proliferative activity within PDO structures, confirming the presence of actively cycling tumor cells. (**B**) Cell viability within PDOs was assessed using Cyto3D live/dead staining. Viable cells were indicated by green fluorescence, whereas non-viable (dead) cells were indicated by red fluorescence, demonstrating the overall cellular viability and structural integrity of the organoids.

After two weeks of culture, PDOs from NB and MBL were embedded and paraffin blocks were cut to obtain 5 μm slices using a microtome. H&E staining was performed in order to histologically characterize the PDOs in accordance with the pathological diagnosis. To further evaluate the PDOs, we evaluated the expression of a commonly employed proliferation marker (Ki-67) by immunohistochemistry. The positive staining of Ki-67 confirmed that PDOs retained the histological tumor features (Figure 5A). Furthermore, we evaluated the compatibility of cell viability assays on organoid models. After growing the NB (9464D-M2) organoids for 7–10 days, we performed a cell viability assay by using Cyto3D and observed live and dead cells in organoids by using the Sartorius Incucyte system (Figure 5B).

## 4. Discussion

Traditional 2D culture systems have played an important role in pediatric cancer research; however, their limited ability to recapitulate tumor architecture, developmental context, and immune complexity significantly constrain their translational utility. Pediatric organoids are used to study tumor biology, including developmental pathways (Notch, WNT, SHH, TGF-beta) and fusion-driven oncogenesis that are characteristic of childhood cancers [27]. Given the unique biology of childhood cancers, often driven by developmental dysregulation rather than high mutational burden, PDOs provide a biologically relevant platform to interrogate patient-specific disease mechanisms and therapeutic vulnerabilities.

Over the past 20 years, advances in stem cell biology and in vitro 3D culture have heralded a revolution in biology and medicine. A major recent step in this revolution has been the development of methods to generate, under controlled cultured conditions, 3D structures, known as organoids [28]. Among the multiple organoid applications, the establishment of cancer organoids has recently emerged as a prominent tool to enhance our understanding of human cancers by faithfully mimicking in vitro both inter- and intra-tumoral heterogeneity [29,30,31]. In preserving patient-specific tumor genetics and enabling co-culture with immune cells, fibroblasts, vasculature and intracellular matrix components, organoids will allow investigators to elucidate immune therapy effects in a controlled yet biologically active meaningful setting. These systems can model tumor–immune interactions, immune evasion mechanisms, cytokine signaling and checkpoint pathway effects, creating an understanding of why tumors respond, or fail to respond, to immunotherapies.

Patient-derived organoids with their ability to be expanded in vitro while preserving many features of primary tumors offer an interesting opportunity to perform studies requiring both high sample quality and quantity. Patient-derived organoids arising from human patients can be directly established from tumor needle biopsies [32], surgical resections [33], bone marrow aspirates, CSF or ascitic and pleural fluid [34] to perform “top-down” studies of pre-established malignancies. They are of particular interest for rare cancers, especially in pediatrics, as they allow the generation of large collections of living material for research purposes, despite the scarcity and small tumor sample sizes.

Organoid models appear to be ideal models to study how tumoral heterogeneity impacts response to treatments, which has barely been investigated, so far, in childhood cancers. As they depict inter-tumor heterogeneity, pediatric tumoroid models should enable us to understand why patients that apparently present with the same clinical features respond differently to treatments, a key issue in therapeutic management. Moreover, they should clearly define how intra-tumor cell heterogeneity and plasticity contribute to resistance to treatments and relapses. Indeed, by allowing the amplification of tumor material while preserving its characteristics as closely as possible, organoids may allow unprecedented single-cell-omics approaches to unravel, at genetic–epigenetic–transcriptomic levels, the diversity of tumor cells before and after treatment. Moreover, the relative ease of genetically modifying them to express fluorescent reporters of specific tumoral sub-populations [34,35] should help to dissect and monitor, over time and by 3D imaging [36], the mechanisms identified as playing a causal role in tumor escape.

## 5. Conclusions

In conclusion, we were able to generate pediatric cancer PDOs, an alternative tumor preclinical model exhibiting the features of the diverse spectrum of pediatric solid tumors. PDOs preserved key histological features and lineage-specific marker expression, and recapitulated tumor tissue heterogeneity and the immune microenvironment. The developed organoid models may be used for preclinical studies that will establish a robust foundation for the translation of patient-derived organoid-guided therapeutic strategies into early-phase clinical trials for pediatric cancers. Findings from organoid-based drug screening and validation in preclinical xenograft models will inform the selection of prioritized therapeutic regimens for clinical evaluation and advance precision medicine approaches, leading to personalized treatment options for children with rare and aggressive malignancies.

## 6. Limitations and Future Directions

Despite the advantages of patient-derived organoids (PDOs) as physiologically relevant in vitro models, several limitations should be considered. PDOs lack functional vasculature, which restricts the accurate modeling of nutrient gradients, drug delivery, and tumor–endothelial interactions. Additionally, stromal and microenvironmental components, including fibroblasts and immune cells, may be progressively lost during long-term culture, potentially reducing the complexity of tumor–microenvironment interactions. The establishment and maintenance of organoid cultures are also associated with relatively high costs and technical demands, which may limit scalability for large-scale screening applications. Furthermore, while PDOs recapitulate key tumor characteristics, their predictive value requires validation in orthotopic in vivo models to fully assess therapeutic responses. Future studies should focus on integrating PDOs with co-culture systems, such as autologous immune cells or stromal components, and combining these models with in vivo validation to enhance their translational applicability.

## Data Availability

The data presented in this study are available on request from the corresponding author due to privacy and ethical restrictions related to patient-derived samples.

## Supplementary Materials

The following supporting information can be downloaded at https://www.mdpi.com/article/10.3390/cancers18091465/s1: Figure S1: Organoid size was quantified on day 1, day 5, and day 10 using ImageJ software. Figure S2: Organoids generated from bone marrow aspirates of neuroblastoma (NB) and Ewing sarcoma (EWS) were measured using ImageJ software.
