# Supplementary material for: Establishment of Patient-Derived Organoids for Pediatric Cancer Research
Source: Cancers (Basel). 2026 May 2;18(9):1465. doi: 10.3390/cancers18091465 (PMC13162899; doi:10.3390/cancers18091465)
Supplement: Supplementary file 1 [file cancers-18-01465-s001.zip › cancers-4280192-supplementary/cancers-4280192-suppl. resub/cancers-4280192-suppl. resub/cancers-4280192 author resub2 suppl..pdf]

Supplemtry File

Establishment of Patient-Derived Organoid for Pediatric Cancer Research

Muhammad Younis[1], Tarlan Arjmandi[1], Mohammad Haque[1], Katherine McClain[1], Thussenthan Walter-Angelo[1], Franklin Back[1], Divya Gandra[1], Abigail Moore[1], Chandrika Behura[1,2], Vladimir Spiegelman[1], Hong-Gang Wang, Sunisa Dovat, Jeremy Hengst[1*], Giselle Saulnier Sholler[1,2*]

[1]Department of Pediatrics, Hematology/Oncology, Pennsylvania State University, College of Medicine, Hershey, PA, USA 17033

[2]Pediatric Hematology and Oncology Division, Pennsylvania State University, Golisano Children's Hospital Hershey, PA, USA 17033

Correspondence and requests for materials should be addressed to

*Giselle Saulnier Sholler and Jeremy Hengst

Email: gsaulniersholler@pennstatehealth.psu.edu

Email: jhengst@pennstatehealth.psu.edu

Phone: (802) 338-0703

Phone: (717) 3850687

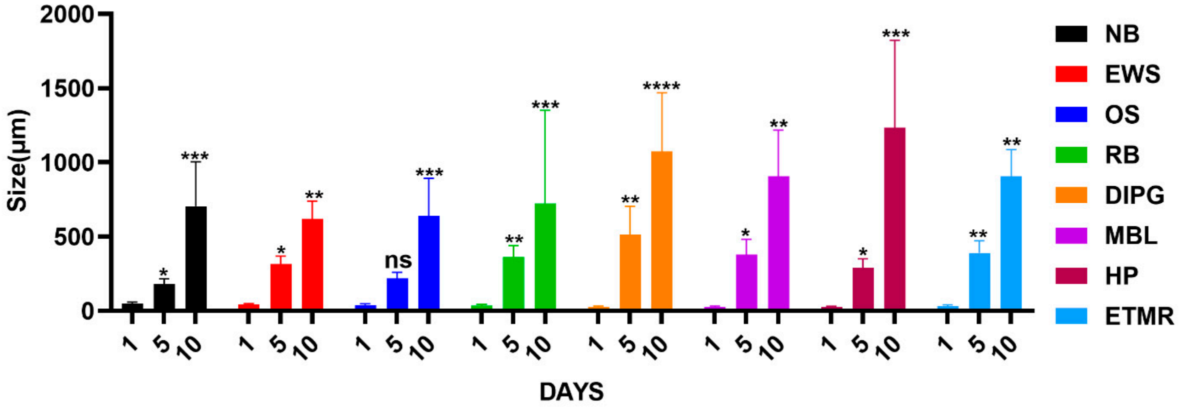

Figure S1. Organoid size was quantified on day 1, day 5, and day 10 using ImageJ software. Data are presented as mean ± SD. Statistical analysis was performed using [test name], with significance indicated as ns = not significant., $p < 0.05$, *$p < 0.01$, **$p < 0.001$.

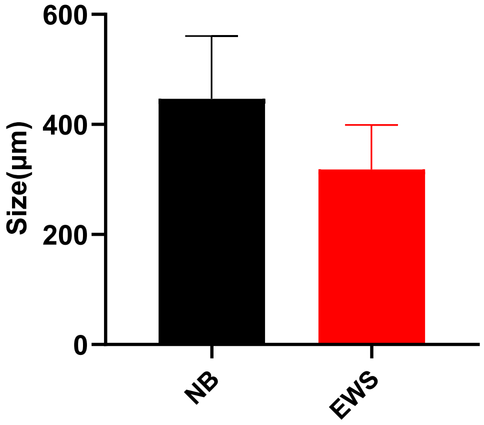

Figure S2. Organoids generated from bone marrow aspirates of neuroblastoma (NB) and Ewing sarcoma (EWS) were measured using ImageJ software.
