# Supplementary figures and images for: Establishment of Patient-Derived Organoids for Pediatric Cancer Research
Source: Cancers (Basel). 2026 May 2;18(9):1465. doi: 10.3390/cancers18091465 (PMC13162899; doi:10.3390/cancers18091465)

PCNA

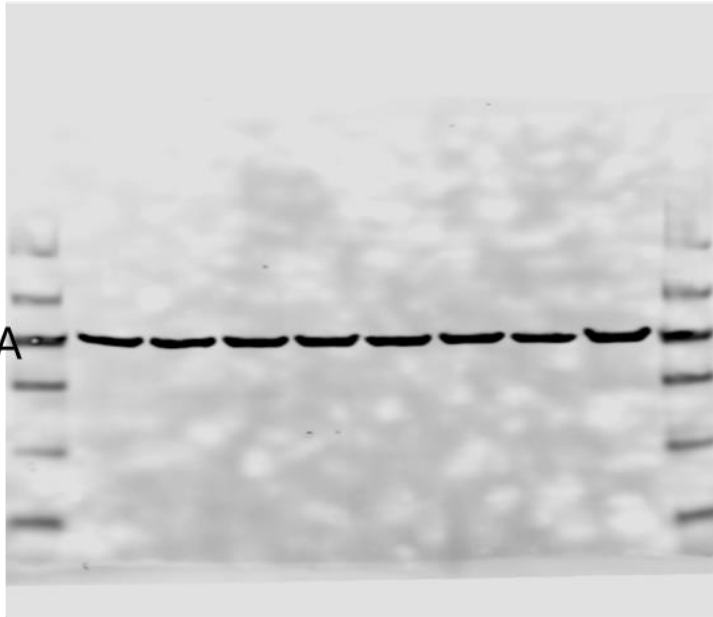

Vimentin

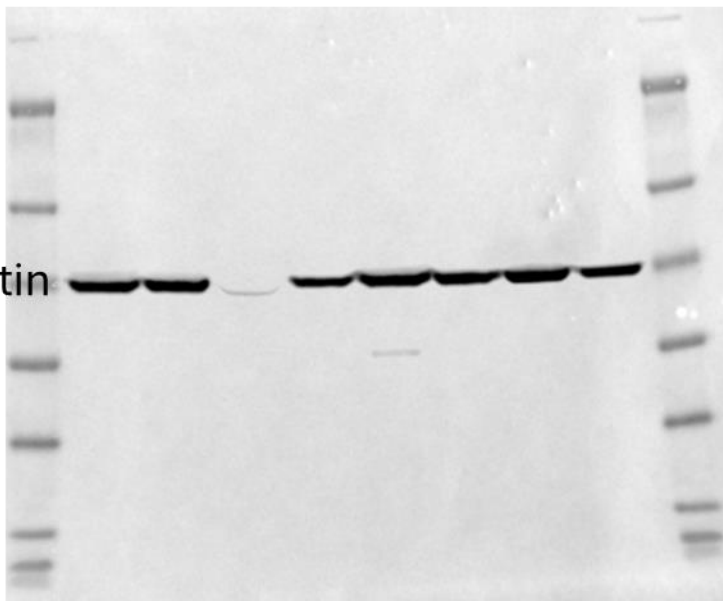

N-myc

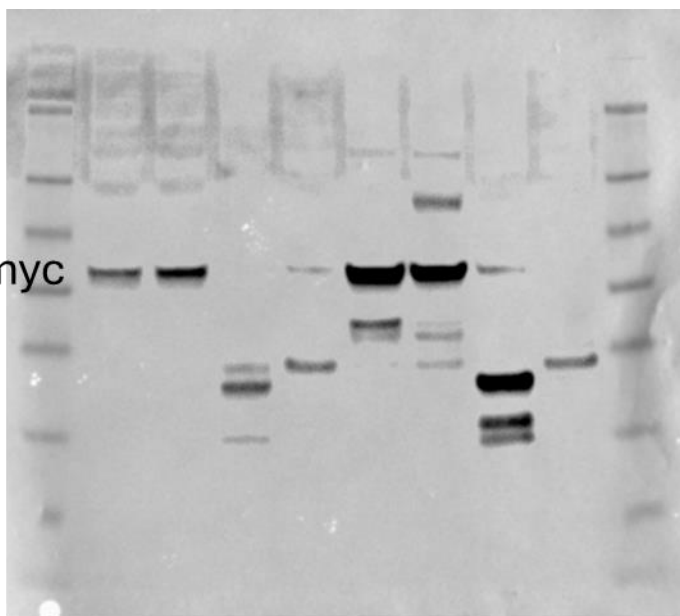

Sox-2

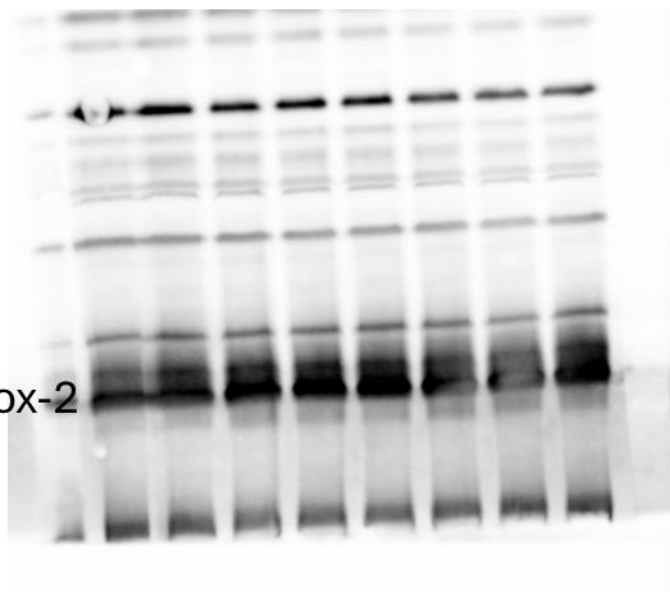

NANOG

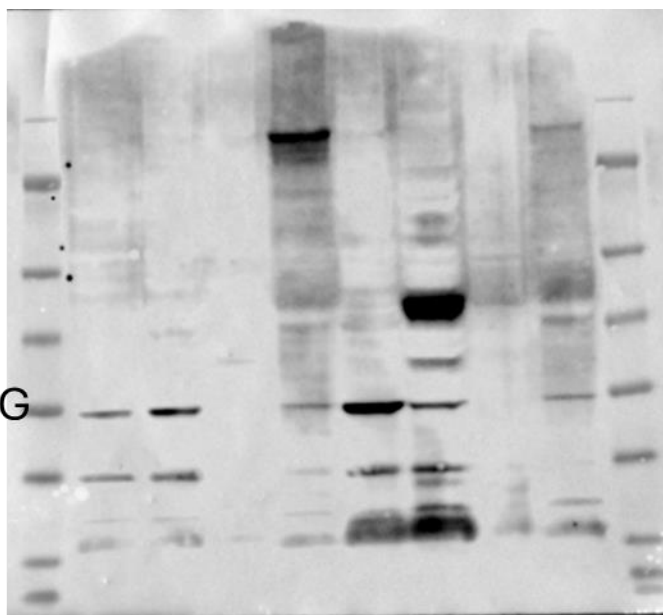

GAPDH

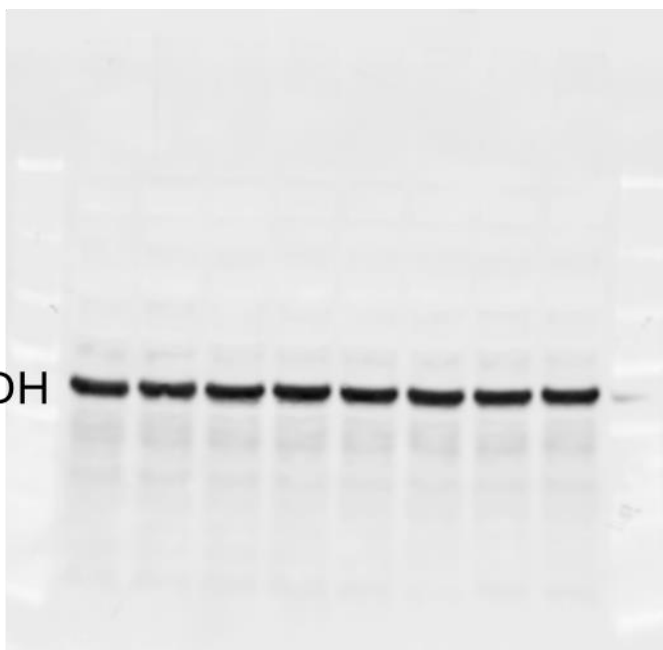

Supplement: Supplementary file 1 [file cancers-18-01465-s001.zip › cancers-4280192-supplementary/cancers-4280192-suppl. resub/cancers-4280192-suppl. resub/cancers-4280192-WB.pdf]
